# A Biochemical View on Intermittent Fasting’s Effects on Human Physiology—Not Always a Beneficial Strategy

**DOI:** 10.3390/biology14060669

**Published:** 2025-06-09

**Authors:** Willian F. Zambuzzi, Marcel Rodrigues Ferreira, Zifan Wang, Maikel P. Peppelenbosch

**Affiliations:** 1 Laboratory of Bioassays and Cellular Dynamics, Department of Chemistry and Biochemistry, Institute of Biosciences, UNESP—São Paulo State University, Botucatu 18618-970, São Paulo, Brazil; w.zambuzzi@unesp.br (W.F.Z.); marcelrodrigues45@yahoo.com.br (M.R.F.); 2 Key Laboratory of Biotechnology and Bioengineering of State Ethnic Affairs Commission, Biomedical Research Center, Northwest Minzu University, Lanzhou 730030, China; zifanwang0316@gmail.com; 3 China-Malaysia National Joint Laboratory, Northwest Minzu University, Lanzhou 730030, China; 4 Department of Gastroenterology and Hepatology, Erasmus MC—University Medical Center Rotterdam, P.O. Box 2040, NL-3000 CA Rotterdam, The Netherlands

**Keywords:** intermittent fasting, insulin sensitivity, molecular mechanism, AMPK, microbiome

## Abstract

Intermittent fasting (IF) has emerged as a widely practiced dietary regimen, increasingly utilized in both clinical and non-clinical settings for its potential health benefits. Evidence suggests that IF can improve metabolic health by enhancing insulin sensitivity, reducing inflammation, and aiding weight management. Recent studies have also explored its role in mitigating obesity-related diseases, such as type 2 diabetes and non-alcoholic fatty liver disease, and its ability to support cardiovascular health and mental function. The effects of IF, however, vary depending on individual health conditions. Some patients show no clinical improvement, while others experience worsened outcomes. Mechanistically, IF induces metabolic switching and activates adenosine monophosphate-activated protein kinase (AMPK), both of which contribute to its therapeutic potential. These responses are influenced by factors such as underlying pathology, baseline metabolic state, and dietary composition. While preclinical data indicate potential therapeutic effects in diseases like cancer, rheumatoid arthritis, and neurodegenerative conditions, these findings are not yet sufficiently supported by human studies. This review argues that IF holds promise as a disease-modifying intervention. However, its implementation should be personalized according to patient-specific characteristics, and future clinical trials must prioritize identifying optimal fasting protocols to maximize therapeutic outcomes.

## Simple Summary

Intermittent fasting (IF) has gained popularity both as a dietary practice and as a potential therapeutic approach in clinical settings. While widely used for health benefits such as weight management and improved insulin sensitivity, its effects on various diseases remain complex. Studies suggest IF could influence metabolic processes, alleviate obesity-related conditions like type 2 diabetes, and improve mental health, but responses vary significantly across individuals. Recent research highlights its potential in treating diseases like non-alcoholic fatty liver disease, cardiovascular conditions, and cancer, though its long-term effects are still under investigation. The mechanisms behind IF, including the activation of metabolic pathways like AMPK, may offer insights into how it can be more effectively applied in specific patient groups. However, the evidence supporting IF as a universal disease-modifying strategy is limited, and many proposed trials may lead to disappointing results. This review argues that the therapeutic potential of IF should be carefully considered, with future research focusing on optimizing protocols for individual needs and specific pathologies.

## 1. Intermittent Fasting Is Increasingly Used in a Medical/Clinical Context

Intermittent fasting constitutes a voluntary temporal abstinence or reduction in food consumption, usually according to a pattern or rhythm. These rhythms partially follow circadian principles but may be modified by external or internal disruptions, such as feeding schedules that do not align with the natural light–dark cycle. These are referred to as (semi-)circadian rhythms [1]. Being a popular form of dieting, intermittent fasting is widely practiced for a variety of societal or religious reasons (e.g., Ramadan), but also increasingly for its perceived health benefits [2]. With respect to the latter, a substantial body of research has shown that intermittent fasting exerts a wide spectrum of impacts on improving indicators of health, especially with regard to weight management [3], while recent meta-analyses support its effects on insulin sensitivity [4] and inflammation [5], even though a recent study failed to replicate the effect of time-restricted eating over calorie restriction per se on metabolic health in obese individuals [6]. Intriguingly, preclinical work in experimental rodents [7] supports that intermittent fasting is associated with a prolonged life expectancy, although there is a paucity of data that corroborate that similar effects exist for humans as well [8]. A recent study in elderly human subjects, however, showed improved mental faculties following intermittent fasting [9]. Thus, the body of evidence that supports intermittent fasting as a healthy life choice appears compelling.

Not surprisingly, the notion that intermittent fasting supports health has led to studies investigating the potential use of intermittent fasting as a disease-modifying strategy. While short-term studies certainly support improved clinical behavior of obesity-related diseases like type 2 diabetes [10] and non-alcoholic steatotic liver disease [6], beneficial responses to intermittent fasting are certainly not universal, with a substantial fraction of patients actually showing a worse clinical course when submitting themselves to intermittent fasting [11], while a recent meta-analysis showed that apart from increases in high-density lipoproteins, intermittent fasting had no significant long-term effects on insulin, hemoglobin A1c%, total cholesterol, low-density lipoprotein, or systolic blood pressure in obesity [12]. In oncological medicine, intermittent fasting has been linked to improved responses to advanced chemotherapeutic regimens like FOLFOX [13], while trials investigating its potential for improving quality of life when subjected to such therapies are currently being conducted as well [14]. Also, in rheumatoid arthritis and asthma, positive effects of intermittent fasting have been reported [15] and further trials have been initiated [16]. Similarly, trials in autoimmune disease like inflammatory bowel disease [17,18] and lupus [19] are contemplated as well. Prompted by encouraging results in mice, such studies are proposed in Parkinson’s disease [20] and stroke [21,22] as well. In animal studies, the improvements in cardiovascular health indicators show up at 2–4 weeks after the start of intermittent fasting [23] and a clinical trial in myocardial infection has been initiated [24]. Hence, it is fair to say that a broad variety of pathologies will be investigated in the near future for their potential to be clinically targeted through intermittent fasting.

Here, however, we shall argue through exploring the biochemical effects of intermittent fasting, suggesting that its effects may vary across individuals and conditions, and are possibly more pronounced in certain patient populations or pathological contexts. As insights into the mechanisms mediating the effects of intermittent fasting beyond calorie restriction slowly emerge, it should become possible to identify specific individuals likely to benefit from intermittent fasting and also to develop more effective fasting protocols. Overall, we shall argue that many of the currently proposed trials are likely to yield disappointing results.

## 2. Influence of Diet Composition and Cultural Eating Patterns on Fasting Outcomes

Evidence suggests that the metabolic effects of intermittent fasting are significantly influenced by the quality of the diet consumed during eating windows. The types and quantities of macronutrients, along with cultural dietary contexts, affect inflammatory responses, insulin sensitivity, and gut microbiota. For example, the Mediterranean diet, rich in fruits, vegetables, whole grains, and healthy fats, may enhance the benefits of fasting through its anti-inflammatory properties and improved metabolic outcomes. In contrast, the Western diet, high in saturated fats and refined sugars, may hinder fasting benefits by promoting chronic inflammation and insulin resistance [25,26]. Traditional Eastern diets, low in red meat and sugars, also show favorable metabolic effects but are being impacted by Western dietary influences [27,28] (please, see Table 1). Therefore, the success of intermittent fasting is closely tied to the underlying dietary patterns, highlighting the need for future studies to consider these factors for personalized nutrition.

## 3. Intermittent Fasting and Reset of Metabolic Physiology

Intermittent fasting per se is associated with reduced calorie intake both in experimental animals [29] as well as in human subjects [30], although over time, this decrease in energy intake tends to diminish as adherence decreases, and individuals may develop compensatory behaviors—such as overeating on non-fasting days or reducing physical activity—which can offset the initial benefits [31]. Although in general, the degree of weight loss achieved with intermittent fasting is on a par with that achieved with traditional dieting approaches (daily calorie restriction) [30], vocal intermittent fasting advocates maintain that periodic fasting helps with resetting metabolic processes (so-called metabolic switching) [32], in turn supporting fat utilization and insulin sensitivity. This idea is supported by pointing out an evolutionary perspective. It is maintained, originally based on the Kalahari Research Project data, that primitive Dobe !Kung hunter-gatherers often consume meals twice a week while maintaining a vigorous physiology, even as more recent research has discredited this notion [33]. In addition, it can be argued that as great apes spend most of the day foraging and eating [34], constant food intake has been the evolutionary norm for our species. Regular meals mitigate glucose spikes [35] and have thus been proposed to protect pancreatic beta cells from overstimulation in diabetes [36], a key aspect in long-term metabolic health in these patients. In apparent agreement, in obese subjects, the ingestion of meals in a low-frequency pattern increases postprandial insulin responses [37]. Thus, the beneficial effects of intermittent fasting beyond the effects of calorie restriction do not intuitively emerge from our current insights in the workings of human physiology.

## 4. Intermittent Fasting, Activation of AMPK, and Human Disease

The depletion of (hepatic) glycogen stores will activate adenosine monophosphate-activated protein kinase (AMPK) in metabolically relevant cells [38]. AMPK acts as a key cellular energy sensor, becoming activated when the AMP/ATP ratio increases during energy stress, such as during fasting. Once activated, AMPK promotes catabolic pathways that generate ATP, such as fatty acid oxidation and autophagy, while inhibiting anabolic pathways that consume ATP, including gluconeogenesis and lipid synthesis. In the context of type 2 diabetes, AMPK activation improves insulin sensitivity, enhances glucose uptake in skeletal muscles, and reduces hepatic glucose production, thereby contributing to better glycemic control [39]. Evidence from patients with metabolic syndrome shows that lipolysis can begin as early as four hours after glucose withdrawal, following overnight fasting and the consumption of 70 g of glucose [40]. This suggests that even moderate temporal abstinence from caloric intake may reduce lipid droplet size [41], an effect beneficial in individuals with metabolic dysfunction-associated fatty liver disease (MAFLD/NAFLD) [42,43]. The reduction in hepatic lipid content helps alleviate endoplasmic reticulum stress, which may improve hepatocellular function [44].

Intermittent fasting may also have implications for physically active individuals. In combination with moderate exercise, temporal restriction may facilitate fat loss in athletes who compete in weight-sensitive sports such as judo, boxing, or weightlifting [45]. From approximately 12 h of fasting onward, ketone levels begin to rise in healthy individua [46]. This signals a shift in energy substrate preference, where the body increases reliance on ketone bodies derived from fatty acid oxidation. Although this is generally viewed as metabolically adaptive, it can lead to catabolism of muscle proteins and elevated ketone flux. These effects may impair muscle strength [47] and hinder muscle repair by inducing a quiescent state in muscle stem cells through ketone body signaling [48]. Thus, while intermittent fasting can be beneficial for body composition in athletes, fasting periods longer than 12 h may introduce risks. Monitoring levels of β-hydroxybutyrate and acetoacetate—the main products of hepatic ketogenesis—is recommended when designing fasting protocols for performance-driven contexts [49].

Intermittent fasting-induced ketogenesis may also provide therapeutic benefits for specific clinical populations. Ketogenesis is triggered under conditions of prolonged fasting or carbohydrate restriction, during which insulin levels fall and hepatic fatty acid oxidation increases. As acetyl-CoA from β-oxidation exceeds the capacity of the tricarboxylic acid (TCA) cycle, it is converted into ketone bodies—primarily acetoacetate and β-hydroxybutyrate (BHB). BHB serves as a highly efficient energy substrate for peripheral tissues, including skeletal muscle and the brain, especially under low-glucose conditions. However, sustained ketogenesis may also affect anabolic signaling. Lowered insulin and IGF-1 levels can suppress mTOR activity, impairing muscle protein synthesis and limiting immune cell proliferation—two energetically demanding processes. In patients with steatotic liver disease, repeated, transient ketogenesis appears to be beneficial, potentially through mechanisms involving immune modulation [50,51].

Moreover, ketone body signaling has been shown to upregulate endothelial Oct4 expression, which may improve vascular wall integrity. This cyclic ketogenesis could benefit individuals with compromised vascular function, such as former smokers. A growing body of preclinical evidence suggests that intermittent fasting may help prevent or delay vascular dementia, possibly via both ketosis-dependent and AMPK-dependent mechanisms [52,53].

In the context of intensive care medicine, delayed parenteral nutrition—which induces mild ketosis—has been associated with improved clinical outcomes in both adults [54] and children [55], although not all studies have confirmed these effects [56]. Therefore, while promising, the clinical benefits of intermittent fasting and ketosis in critically ill patients remain inconclusive and require further investigation [57].

Overall, intermittent fasting’s ability to repeatedly activate AMPK represents a powerful mechanism with potential applications in metabolic health, liver disease, vascular function, and beyond. However, translating these benefits into standardized clinical protocols remains a challenge due to the complexity of metabolic responses and individual variability.

### 4.1. Molecular and Biochemical Pathways Underlying Fasting-Induced Metabolic

Intermittent fasting triggers a coordinated metabolic adaptation through pathways regulated by AMPK, mTOR, glycolysis/gluconeogenesis, and fatty acid metabolism. AMPK acts as an energy sensor, activated by increased AMP/ATP ratios during fasting. It shifts metabolism from anabolic to catabolic processes by inhibiting lipid, cholesterol, and protein synthesis while promoting ATP production through fatty acid oxidation and autophagy [58,59].

Fasting also inhibits the mTOR signaling pathway, reducing insulin and nutrient availability, which shifts the focus from growth to maintenance and repair, potentially extending lifespan. It modulates glucose metabolism by decreasing glycolytic flux and inducing gluconeogenic enzymes to preserve glucose for essential tissues [60,61,62].

Fatty acid metabolism becomes crucial during fasting as hormone-sensitive lipase promotes lipolysis, releasing fatty acids into circulation. AMPK further enhances mitochondrial β-oxidation and ketone body production from acetyl-CoA, providing energy for other tissues. Overall, intermittent fasting induces biochemical reprogramming that enhances oxidative metabolism, autophagy, and energy source mobilization, supporting survival during nutrient scarcity and highlighting its therapeutic potential for metabolic and age-related conditions [60,63,64].

### 4.2. Intermittent Fasting in Athletes: Benefits, Risks, and Metabolic Trade-Offs

Athletes represent a unique subgroup in which the application of intermittent fasting requires careful consideration. While the metabolic principles of fasting—such as enhanced fat oxidation and improved insulin sensitivity—can be advantageous for body composition and weight management, the same adaptations may pose risks for muscle preservation and recovery. In sports with weight categories, such as judo, boxing, or wrestling, moderate temporal restriction combined with structured training can reduce fat mass and improve lean mass ratios. Evidence suggests that even short fasting windows, when strategically applied, promote early lipolysis and reductions in visceral fat, aiding in weight regulation without compromising performance [65].

However, fasting durations exceeding 12 h may induce ketosis, increasing circulating levels of ketone bodies such as β-hydroxybutyrate. While these compounds serve as alternative energy substrates, they can also signal muscle stem cells into a deeply quiescent state and downregulate mTOR signaling, thereby impairing muscle protein synthesis and regeneration. This phenomenon may be particularly detrimental when muscle repair is required after high-intensity training or injury. Moreover, prolonged fasting may also elevate muscle protein catabolism as amino acids are mobilized for gluconeogenesis and energy production in late fasting phases [64].

These dual effects highlight the complexity of intermittent fasting in athletic populations. Unlike patients with metabolic syndrome—where AMPK activation and ketogenesis are predominantly beneficial—athletes must maintain a delicate balance between metabolic efficiency and anabolic demands. As such, fasting protocols for athletes should be individually tailored, with attention to fasting duration, training load, and recovery needs. Monitoring biomarkers such as circulating ketones may help ensure that metabolic adaptations remain within a physiological range that supports, rather than hinders, performance.

The depletion of (hepatic) glycogen stores will activate adenosine monophosphate-activated protein kinase (AMPK) in metabolically relevant cells [38]. AMPK acts as a key cellular energy sensor, becoming activated when the AMP/ATP ratio increases during energy stress, such as fasting. Once activated, AMPK promotes catabolic pathways that generate ATP, such as fatty acid oxidation and autophagy, while inhibiting anabolic pathways that consume ATP, including gluconeogenesis and lipid synthesis. In the context of type 2 diabetes, AMPK activation improves insulin sensitivity, enhances glucose uptake in skeletal muscle, and reduces hepatic glucose production, thereby contributing to better glycemic control [39]. Studies in patients with metabolic syndrome suggests that following consumption of 70 g of glucose after an overnight fast, lipolysis commences already four hours after the onset of fasting [40] and thus even moderate temporal abstinence of calorie intake results in reduced size of the lipid droplet compartment [41], an event beneficial in individuals with for instance metabolic dysfunction-associated fatty liver disease/non-alcoholic fatty liver disease [42], mainly because the resulting reduction in steatosis relieves hepatic endoplasmic cell stress [44]. Likewise, especially in combination with moderate exercise, moderate temporal restriction may aid in losing fat for athletes competing in sports with weight categories [45], like, e.g., Judo, boxing, or weight-lifting. From 12 h of fasting onwards, however, in healthy controls, blood ketone starts to increase [46], and thus, body protein starts to be converted to energy, potentially negatively affecting muscle strength through increased muscle ketone flux [47], while concomitantly provoking deep quiescence in muscle stem cells through ketone body signaling [48], hampering muscle repair following (exercise-induced) damage. Hence, intermittent fasting regimes aimed at improving athlete anthropomorphic characteristics probably require careful design (fasting periods should probably be shorter as 12 h) and monitoring (for ß-hydroxybutyrate and acetoacetate, the main primary products of hepatic ketogenesis [49]), so as to avoid ketone flux.

Intermittent-fasting-provoked ketogenesis may have additional benefits for specific groups as well. Ketogenesis is triggered during prolonged fasting or carbohydrate restriction when insulin levels drop and hepatic fatty acid oxidation increases. Under these conditions, acetyl-CoA derived from β-oxidation exceeds the capacity of the tricarboxylic acid (TCA) cycle and is diverted to form ketone bodies—primarily acetoacetate and β-hydroxybutyrate (BHB). BHB acts as an efficient alternative energy substrate for peripheral tissues, including muscle and brain, especially during periods of low glucose availability. However, prolonged ketogenesis may also influence anabolic processes: low insulin and IGF-1 levels can reduce mTOR signaling, thereby impairing muscle protein synthesis and limiting immune cell proliferation, which are both energetically demanding processes. In the clinical context, ketogenesis appears beneficial in steatotic liver disease (although the mechanisms involved remain obscure; immune modulation potentially plays a role [50,51]), supporting the role of intermittent fasting in managing such patients through repeated temporary ketogenesis that might be uncoupled from the effects on calorie intake per se. In addition, ketogenesis improves vascular wall condition through the post-translational upregulation of endothelial Oct4 expression, and hence, the cyclic ketogenesis associated with intermittent fasting might be beneficial for subjects with poor vessel condition, e.g., (former smokers), and indeed a body of preclinical evidence supporting the usefulness of intermittent fasting for counteracting vascular dementia exists (e.g., [53]), although such effects may have a ketosis-independent but AMPK-dependent component as well [52]. In intensive care medicine, inducing ketosis by delaying parenteral calorie intake is associated with improved outcomes, both in adults [54] as well as children [55], even though not all studies fully recapitulated this result [56] and the results remained mixed. Thus, the potential benefits of intermittent fasting and in ketosis, in general, for such patients remain uncertain and future work is needed to establish whether such a benefit exists [57]. Generally speaking, although the capacity of intermittent fasting to repeatedly activate AMPK may be exploited for improving human health in specific situations, it remains very difficult to translate this notion into specific protocol recommendations.

## 5. Potential Effects of Intermittent Fasting via AMPK-Mediated mTOR Inhibition on Cancer and Immunity

Apart from its effects on metabolic state, the activation of AMPK can influence processes relevant for oncological medicine. As the activation of AMPK slows down mitotic processes in pre-cancerous tissue [66], the potential for malignant transformation arising from such tissue is diminished. In addition, through reduced activity of signaling through the Mammalian Target of Rapamycin (mTOR), AMPK stimulates cellular autophagy and some cancer cells appear to be specifically sensitive to increased autophagy, counteracting the oncological process, and thus, submitting themselves to intermittent fasting might constitute rational lifestyle advice in patients at risk for oncological disease [67], although in the absence of further proof, e.g., by submitting tumor-prone individuals to intermittent fasting and correlating the effects on AMPK or mTOR activation to the tumor formation (which has been used to objectify the chemopreventive effects of statin treatment through mTOR inhibition in colorectal cancer [68]), such statements remain premature. Intriguingly, however, Every-other-day feeding extends the lifespan in mice by delaying life-limiting neoplastic disorders, and thus, this possibility deserves urgent attention [69].

The biochemical rationale for these effects involves the dual regulation of mTORC1 by AMPK and insulin signaling. Under nutrient-rich conditions, mTORC1 promotes anabolic processes such as protein synthesis and cell proliferation while inhibiting autophagy. During fasting, however, the drop in insulin and activation of AMPK converge to inhibit mTORC1 activity, which in turn triggers autophagy [51,69,70,71]. In cancer biology, enhanced autophagic flux can impair tumor growth by degrading misfolded proteins, damaged mitochondria, and nutrient sources that would otherwise support proliferation. This autophagy-mediated suppression is particularly effective in cancer types that rely on defective proteostasis. Conversely, in immune cells, mTOR activity is required for clonal expansion, cytokine production, and effector differentiation. Thus, the same molecular cascade that protects against malignancy may, under certain conditions, compromise host defenses against infections or impair immune cell function [72].

In this context, it should also be noted that an AMPK-mediated inhibition of mTOR may negatively influence human immunity. The adequate activation of mTOR has emerged as being critical for defending the body against a variety of potentially dangerous viruses, including, e.g., Rotavirus [73] and Hepatitis E [74]. The potential effects of intermittent fasting on the capacity of the body to defend itself against pathogens have not been well-investigated, but in experimental tuberculosis, the immune responses against the mycobacterium have been critically impaired [75], whereas many immune parameters indicate the reduced activity of the immune system in mice subjected to every-other-day feeding [69], and AMPK activation in fasting causes monocytes to reenter the bone marrow, hampering peripheral immune responses [76]. Overall, intermittent fasting can be expected to counteract effective immunity and should not be recommended to patients at high risk of being exposed to infectious agents, e.g., vulnerable individuals commuting using public transport [77].

The reduced functionality of the immune system through the AMPK-mediated inhibition of mTOR signaling in intermittent fasting, however, may be exploited to counteract autoimmune conditions or other pathologies associated with immune system activation. It has, for instance, been reported that intermittent fasting improves factors that are associated with the onset and progression of multiple sclerosis, especially brain-derived neurotropic factor, through the activation of AMPK [78,79]. A recent feasibility study of intermittent fasting in patients with this disease yielded some positive signals, even though these did not reach statistical significance, maybe because the study appeared to be underpowered [80]. It is important to note, however, that despite AMPK and mTOR being highly druggable master regulators of human immunity [81,82,83], the relationship between the pathogenesis of autoimmune disease and AMPK-dependent mTOR inactivation is certainly not clear cut and not many examples exist of successfully targeting human autoimmune disease through the pharmacological targeting of either AMPK or mTOR in the body of contemporary biomedical literature [84,85]. In apparent agreement is the fact that in preclinical models, although intermittent fasting has been used successfully to attenuate experimental autoimmune disease, successful human trails are scarce. An important characteristic of the AMPK-dependent inhibition of mTOR is the induction of autophagy, and defects in autophagy are certainly an important causative factor for many forms of intermittent fasting [86]. Genetic polymorphisms predisposing individuals to autoimmune disease, however, tend to negatively influence autophagy downstream of mTOR, with the *ATG16L1* and *IRGM* polymorphisms predisposing individuals to Crohn’s disease being a good example of this [87]. Overall, employing intermittent fasting to improve the natural history of autoimmune diseases may very well require the definition of very specific patient groups.

## 6. Genetic Variation in the Regulation of AMPK/mTOR Pathways: Implications for Fasting Response

Emerging evidence indicates that genetic background, including ancestry-associated polymorphisms, significantly influences nutrient-sensing pathways like AMPK and mTOR. These pathways are vital for regulating energy metabolism, growth, autophagy, and inflammation, and are involved in responses to intermittent fasting and caloric restriction. For example, in breast cancer cohorts, AMPK pathway enrichment correlates with overall survival in an ancestry-specific manner, showing differences between African American (AA) and European American (EA) patients. Additionally, genetic polymorphisms in the mTOR pathway are linked to cancer risk and hormone receptor status in AA women, suggesting that variations in these genes may affect disease susceptibility and metabolic flexibility [88,89].

Studies in humans have also revealed that genetic and epigenetic factors related to obesity may impair AMPK responsiveness to fasting. In one study, fasting increased LKB1 expression—an upstream kinase of AMPK—and activated AMPK signaling in lean individuals, but this response was blunted in obese participants, despite similar levels of AMPK subunit expression [90]. These findings suggest that obesity may serve as a proxy for underlying genetic or epigenetic regulation affecting AMPK function.

The role of AMPK in immune regulation has also been shown to vary according to genetic background. In monocyte-derived macrophages, decreased AMPK activity leads to metabolic reprogramming and increased mitochondrial dysfunction through the activation of the mTOR/PGC1α axis. This contributes to a pro-inflammatory phenotype, which has been implicated in diseases such as metabolic-associated steatotic liver disease [91]. Notably, genetic variants in AMPK subunits (e.g., PRKAB1) can influence this inflammatory response by altering NF-κB signaling [91].

In support of the role of genotype in modulating dietary responses, a large-scale study using genetically diverse outbred mouse populations demonstrated that the physiological effects of intermittent fasting and caloric restriction are highly dependent on genetic background. Specific gene–diet interactions were identified, including loci affecting cardiac function and metabolic outcomes [92]. These findings underscore the importance of accounting for host genetics in the interpretation of fasting-induced health effects, particularly via the AMPK and mTOR pathways.

Taken together, these data highlight that responses to intermittent fasting are not uniform across individuals and are substantially shaped by genetic variation, including ancestry-associated differences (Table 2). As dietary interventions gain traction in clinical and preventive settings, incorporating genetic profiling may help optimize protocols and identify subgroups that are more likely to benefit—or to experience adverse responses—due to specific AMPK/mTOR pathway configurations.

## 7. Intermittent Fasting May Drive the Acquisition of Oncogenic Mutations or Confer Protective Effects Depending on the Individual Context

The most frequently mutated oncogene in cancer is *KRAS* [94]; for instance, it is mutated in, for practical purposes, all cases of pancreatic ductal adenocarcinoma [95]. Oncogenic KRAS alters the metabolism of tumor cells, resulting in increased glucose uptake and enhanced glycolysis, even in the presence of abundant oxygen (the so-called Warburg effect or aerobic glycolysis [96]). These metabolic effects of oncogenic KRAS have been explained by the transcriptional upregulation of glucose transporters *GLUT1* and *GLUT3* and by stimulating the activity of glycolytic enzymes through transcriptional and other mechanisms [97,98,99]. Importantly, low extracellular sugar drives the expansion of cellular compartments that are more proficient in glucose uptake and mobilization for ATP production [100] and thus provide positive selection pressure in the body for cells harboring oncogenic *KRAS* mutations, increasing the risk for full malignant transformation. In healthy volunteers, even moderate intermittent fasting (9 h per diem in a so-called early time-restricted feeding design involving fasting between 6:00 h to 15:00 h) lowered blood glucose levels by approximately 10% to 25% [101]. Prolonged exposure of cells in the body to such low glucose levels may foster the acquisition of Ras mutations. Especially in the context of other potentially premalignant mutations, a Ras mutation may be sufficient for the progression towards an aggressive cancerous phenotype. For instance, the potential of *KRAS* mutations to confer a metastasis-prone phenotype in the context of colon cancer has been well-described and is non-controversial [102]. Other studies document that lower extracellular nutrient availability can drive the selection of clones that are potentially more carcinogenic, like acquiring alterations in open chromatin upon adaptation to lower external glucose in pancreatic ductal cells [103,104]. One can thus envision that in patients who exhibit relatively large compartments of *KRAS* mutation-negative premalignant cells, e.g., patients with an extended Barrett’s segment in the esophagus [105,106] or substantial intestinal metaplasia in the stomach [107,108], intermittent fasting does not constitute a rational strategy in the management of such individuals. Indeed, whereas in general weight loss intermittent fasting would be expected to confer protection from gastric cancer development [109], in a large prospective multicenter cohort study body weight loss was not associated with reduced propensity to progression from intestinal metaplasia to full-blown gastric cancer [110].

Having pointed out that intermittent fasting is not necessarily indicated in the management of individuals with potentially pre-cancerous conditions, the weight loss associated with intermittent fasting per se should have a preventive effect with regard to oncological disease in general. Overweight or obesity is a strong risk factor of cancer incidence at several cancer sites [111]. Especially in colon cancer, a relationship between obesity and fat mass is evident [112], potentially because the fat-derived hormone leptin is potent growth factor for pre-cancerous cells in the colon [113]. Importantly, intermittent fasting reduces leptin levels substantially, especially when combined with physical activity [114], although there is a notable trend showing that this effect is weaker in more recent studies [115] as compared to older studies [116]. Furthermore, there is uncertainty on the capacity of intermittent fasting to maintain the reduced size of the adipose compartment—and per extenso leptin production—with a recent systematic review highlighting the possibility that the weight loss efficacy of time-restricted eating without calorie counting may peak around 3 months [117], which is probably not enough to significantly dent the life time risk for developing adiposity-related cancer. Also, the efficacy of intermittent fasting over calorie restriction per se for controlling adiposity remains uncertain, although an effect may be present [118]. Thus, while controlling adiposity is an important goal for improving health, in general, and also in the context of oncological disease, the specific additive value of intermittent fasting for controlling cancer development though effects on fat mass is for now far from obvious and requires further research at best.

## 8. Effects of Time-Restricted Eating Through the Modulation of the Microbiome

It remains controversial whether intermittent fasting confers specific health benefits or potentially deleterious effects beyond calorie restriction per se [119]. Cochrane systematic reviews and meta-analyses are regarded as the gold standard for high-quality information [120,121]. A Cochrane review on the potential role of intermittent fasting for the prevention of cardiovascular disease found that while intermittent fasting was seen to be superior to ad libitum feeding in reducing weight, this was not clinically significant [122]. A long-term clinical trial on intermittent energy restriction in patients with type 2 diabetes did not evidence the superiority of time-restricted eating over conventional calorie restriction [123]. Compared to conventional calorie restriction, however, intermittent fasting poses risks due to dehydration, hypotension, and safety issues related to hypoglycemia and glycemic variability. Although not investigated in a systematic fashion, case reports suggest that, for instance, Ramadan intermittent fasting can provoke severe hypoglycemia that poses risks to car drivers or those using potentially dangerous apparatus [124]. Hence, intermittent fasting advocates are challenged to come up with mechanistic hypotheses that support time-restricted eating beyond calorie restriction.

Advocates generally provide two answers to such challenges [125]. One involves the circadian rhythm. Feeding/fasting paradigms influence the circadian cycle, with time-restricted feeding aligning with circadian cycle-related gene expression, and thus altering physiological processes, at least in experimental animals [126,127,128]. Intermittent fasting regimes are widely divergent with respect to the timing of fasting and it is certainly possible that because meta-analyses pool studies with contrarian timings for food restriction [129], potential effects are obscured. Indeed, an epidemiological study showed the divergent effects of breakfast and lunch skipping compared to dinner skipping with respect to cardiovascular health [130], but it is fair to say that human studies systematically comparing and contrasting the effects of the divergent timing of intermittent fasting in humans are required before statements in this respect can be confidently made.

The second plausible explanation comes from the effects time-restricted eating may have on the microbiome. It is becoming clear that the community of organisms living on the human body is intimately related to health and disease. The major reservoirs for such organisms are the human gastrointestinal tract, different elements of the gut being colonized by other microflora [131], and especially the colon, which dominates the human microbiome. The microbiome is an important determinant of many aspects of physiology, e.g., the colonization of the gut by bacteria following birth and milk consumption drives the transition from fetal intestinal epithelium to more mature forms. For instance, following the partus, the murine intestine does not yet contain crypts before colonization with bacteria. Instead, proliferative cells are restricted to the epithelium between the villi, and the intervillus epithelium reshapes to form crypts and the associated cell types [132]. This process, potentially mediated through induction of the transcription factor *Blimp1* [133], is mediated by the colonization of the gut by butyrate-producing bacteria [134], the latter being a usually bioactive short chain fatty acid with the capacity to profoundly steer intestinal morphogenetic coding [135]. Many relations between microbiome composition, physiology, and pathophysiology have been established, e.g., during pregnancy, the microbiome composition changes and may alter the clinical course of inflammatory bowel disease or even modulate maternal immunity [136] to help the expecting cope with the challenge of maintaining maternal pathogen responses in the face of the relentless fetal uptake of immunoglobulins from the maternal circulation [137]. The importance of controlling bacterial biofilm colonization of the buccal cavity through oral hygiene for maintaining dental health is well-recognized but may even also be related to preventing right-sided colon cancer [138]. Thus, the relationship between microbiome composition and human health is not in doubt.

Importantly, the microbiome is highly dynamic and intermittent fasting has a pronounced effect on its composition. Short-chain fatty acid-producing bacteria are, compared to other bacterial species, relatively proficient in mucus degradation [139], and in the context of food withdrawal, such bacteria quickly obtain a competitive advantage when other intestinal nutrient sources have been depleted. Many potentially beneficial effects have been linked to short-chain fatty acids; for instance, they improved liver enzymes in healthy volunteers following temporally restricted eating, which has been tentatively linked to increased numbers of butyrate-producing bacteria [140]. Such claims, however, await confirmation in studies involving more relevant groups of the population (e.g., patients with steatotic liver disease) and better definition of the mechanistic details by which butyric acid or other short chain fatty acids are potentially active in this respect (e.g., propionic acid [141,142]) in counteracting hepatocyte damage. Intriguingly, however, evidence has been presented that processes like adipocyte browning (obesity may be caused by lowered brown adipose tissue activity [143]) in response to cold challenge may require butyrate-producing flora [144]. Thus, it is possible that the calorie-restriction-independent effects of intermittent fasting may require its combination with other interventions/conditions, obscuring potential effects in epidemiological studies.

## 9. Conclusions

**Figure 1:**
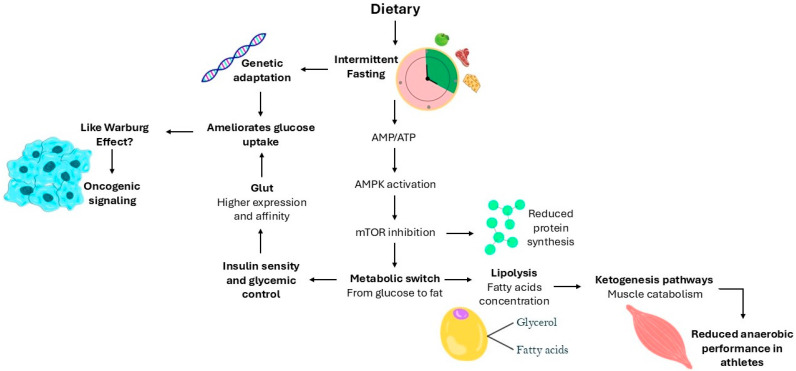
Intermittent fasting-induced metabolic adaptations. The diagram illustrates the metabolic and cellular responses triggered by intermittent fasting. Intermittent fasting increases the AMP/ATP ratio leading to AMPK activation, which subsequently inhibits mTOR signaling. This metabolic switch promotes the transition from glucose to fatty acid utilization, enhancing lipolysis and fatty acid oxidation, while reducing protein synthesis. In the context of prolonged fasting, increased lipolysis leads to the production of fatty acids and glycerol, facilitating ketogenesis. However, excessive ketone production can result in muscle catabolism, compromising anaerobic performance, particularly in athletes. Additionally, prolonged periods of low glycemia can trigger genetic adaptation, increasing the expression and affinity of GLUT transporters. This adaptive response can ameliorate glucose uptake but may also resemble oncogenic signaling, similar to the Warburg effect, as seen in tumor cells.

Overall, intermittent fasting clearly influences physiological processes and can significantly affect the natural history of various diseases. However, whether these effects are beneficial or not is highly context-dependent. The benefits of time-restricted eating compared to traditional calorie restriction per se remain inconclusive, highlighting the need for a better mechanistic understanding of the metabolic and cellular pathways involved. As illustrated in Figure 1, intermittent fasting activates key metabolic pathways, including AMPK signaling, which promote fatty acid oxidation and glucose uptake while inhibiting protein synthesis through mTOR suppression. However, prolonged fasting may also induce ketogenesis, with potential adverse effects on muscle protein preservation, particularly in athletes. Additionally, long-term low glycemia may lead to increased GLUT expression or adaptation to high-affinity glucose transporters, resembling oncogenic metabolic reprogramming. Thus, better biochemical signaling into these processes is crucial to making evidence-based recommendations on the use of intermittent fasting, particularly concerning which individuals are likely to benefit and which may be at risk of adverse outcomes. Personalized fasting protocols that account for genetic background, metabolic state, and lifestyle factors are essential to maximize potential health benefits while minimizing risks.

## Figures and Tables

**Table 1:** Comparison of Mediterranean, Western, and traditional Eastern diets in terms of composition, metabolic impact, and compatibility with intermittent fasting protocols.

Characteristic	Mediterranean Diet	Western Diet	Traditional Eastern Diet
Dominant Components	Fruits, vegetables, whole grains, legumes, olive oil, fish	Red/processed meats, refined grains, processed foods, sugary drinks	Rice, vegetables, soy products, fish, seaweed, tea
Plant-to-Animal Ratio	Highly plant-based, moderate animal intake	Highly animal-based, low plant diversity	Predominantly plant-based, low-to-moderate animal intake
Main Fat Source	Unsaturated fats (olive oil, nuts)	Saturated and trans fats (animal fats, processed oils)	Plant oils, fish-based fats
Fiber and Micronutrient Density	High	Low	High
Portion Sizes/Energy Load	Moderate, nutrient-dense	Large, energy-dense	Moderate, energy-controlled
Impact on Inflammation and Insulin Sensitivity	Anti-inflammatory, improves insulin sensitivity	Pro-inflammatory, promotes insulin resistance	Context-dependent; traditionally protective
Synergy with Intermittent Fasting	High; supports metabolic benefits of fasting	Low; may blunt or counteract fasting effects	Moderate to high; benefits may vary with degree of Westernization
Associated Health Outcomes	Reduced cardiovascular and metabolic disease risk	Increased risk of obesity, diabetes, and CVD	Traditionally associated with longevity and low chronic disease rates

**Table 2:** Summary of key findings on genetic and ancestry-related differences in AMPK/mTOR pathway regulation and their implications for metabolic and inflammatory responses to fasting.

Aspect	Key Findings	Ancestry/Genetic Focus	Reference
AMPK/mTOR in Breast Cancer	AMPK pathway enrichment correlates with OS differently in AA vs. EA patients	Yes (AA vs. EA)	[90]
mTOR Variants and Cancer Risk	mTOR pathway polymorphisms linked to breast cancer risk in African American women	Yes (AA)	[89]
Fasting Response (Lean vs. Obese)	Fasting reduces AMPK activity in lean but not obese individuals; upstream regulation differs	Indirect (obesity as genetic/epigenetic proxy)	[91]
Macrophage Inflammation	AMPK activity modulates inflammatory responses; genetic variation in subunits matters	Yes (subunit variation)	[93]
IF/CR and Genotype in Mice	Dietary intervention effects on physiology depend on genotype; gene–diet interactions mapped	Yes (outbred population)	[92]
